# Psychometric assessment of the Runyankole-translated Marlowe-Crowne Social Desirability Scale among persons with HIV in Uganda

**DOI:** 10.1186/s12889-024-18886-z

**Published:** 2024-06-19

**Authors:** Cristina Espinosa da Silva, Robin Fatch, Nneka Emenyonu, Winnie Muyindike, Julian Adong, Sowmya R. Rao, Gabriel Chamie, Christine Ngabirano, Adah Tumwegamire, Allen Kekibiina, Kara Marson, Brian Beesiga, Naomi Sanyu, Anita Katusiime, Judith A. Hahn

**Affiliations:** 1 Department of Medicine, University of California, San Francisco, CA USA; 2 Faculty of Medicine, Mbarara University of Science and Technology, Mbarara, Uganda; 3 School of Public Health, Makerere University, Kampala, Uganda; 4 School of Public Health, Boston University, Boston, MA USA; 5 Global Health Collaborative, Mbarara University of Science and Technology, Mbarara, Uganda; 6 Infectious Diseases Research Collaboration (IDRC), Kampala, Uganda

**Keywords:** Internal consistency, Reliability, Validity, Measurement error

## Abstract

**Background:**

Social desirability can negatively affect the validity of self-reported measures, including underreporting of stigmatized behaviors like alcohol consumption. The Marlowe-Crowne Social Desirability Scale (SDS) is widely implemented and comprised of Denial and Attribution Domains (i.e., tendencies to deny undesirable traits or attribute socially desirable traits to oneself, respectively). Yet, limited psychometric research has been conducted in sub-Saharan Africa, where the prevalence of unhealthy alcohol consumption is high as well as religiosity and hierarchical social norms. To address this gap, we (a) conducted an exploratory study assessing certain psychometric properties of the 28-item SDS (Runyankole-translated) among persons with HIV (PWH) in Uganda, and (b) examined the relationship between social desirability and self-reported alcohol use.

**Methods:**

We pooled baseline data (*N* = 1153) from three studies of PWH engaged in alcohol use from 2017 to 2021. We assessed the translated scale’s construct validity (via confirmatory factor analysis), internal consistency, item performance, differential item functioning by gender, concurrent validity with the DUREL religiosity index domains, and the association between social desirability and self-reported alcohol use.

**Results:**

Participants had a mean age of 40.42 years, 63% were men, and 91% had an undetectable HIV viral load. The 28-item SDS had satisfactory construct validity (Model fit indices: RMSEA = 0.07, CFI = 0.84, TLI = 0.82) and internal consistency (Denial Domain Ω_Total_ = 0.82, Attribution Domain Ω_Total_ = 0.69). We excluded Item 14 (“I never hesitate to help someone in trouble”) from the Attribution Domain, which mitigated differential measurement error by gender and slightly improved the construct validity (Model fit indices: RMSEA = 0.06, CFI = 0.86, TLI = 0.85) and reliability (Attribution Domain Ω_Total_ = 0.72) of the 27-item modified SDS. Using the 27-item SDS, we found that social desirability was weakly correlated with religiosity and inversely associated with self-reported alcohol use after adjusting for biomarker-measured alcohol use and other confounders (β = -0.05, 95% confidence interval: -0.09 to -0.01, *p*-value = 0.03).

**Conclusions:**

We detected and mitigated measurement error in the 28-item Runyankole-translated SDS, and found that the modified 27-item scale had satisfactory construct validity and internal consistency in our sample. Future studies should continue to evaluate the psychometric properties of the Runyankole-translated SDS, including retranslating Item 14 and reevaluating its performance.

**Supplementary Information:**

The online version contains supplementary material available at 10.1186/s12889-024-18886-z.

## Background

Social desirability (the tendency for someone to present themselves in a generally favorable light) is a common phenomenon globally, although it likely manifests to differing degrees across cultures [1–3]. As an important source of confounding in behavioral health research, social desirability bias in research assessments can negatively affect the validity of self-reported measures [4–7] and has been found to increase underreporting of stigmatized behaviors like alcohol consumption [8–12]. This is particularly relevant in resource-limited settings like sub-Saharan Africa, where the prevalence of alcohol consumption and HIV are high and collecting self-reported data is more feasible compared to costly biospecimens [13, 14]. Moreover, religiosity and hierarchical social norms are prevalent in parts of sub-Saharan Africa, which may influence social desirability trends [15–20].

The Marlowe-Crowne Social Desirability Scale (SDS) is a commonly implemented measure to assess social desirability, and is comprised of two domains: denial (i.e., the tendency to deny undesirable characteristics that one has or, in other words, the tendency to avoid disapproval) and attribution (i.e., the tendency to attribute or amplify socially desirable characteristics to oneself or, in other words, the tendency to seek approval) [3, 21]. While it was developed among undergraduate students in the United States [21], it has been implemented in many settings and populations globally [22, 23]. In sub-Saharan Africa, the original 33-item SDS [21] was pilot-tested in different local languages in Uganda, Kenya, Ethiopia, and Mozambique [24]. Although the languages of the translations were not specified by the authors, they found that the original scale could be shortened to a 28-item scale to exclude items that were not as relatable to participants in their study settings in sub-Saharan Africa (example of omitted item: “I never make a long trip without checking the safety of my car”). Moreover, a study in Uganda using the 13-item Marlowe-Crowne SDS Short Form C found that social desirability was inversely associated with self-reported alcohol consumption among persons with HIV (PWH) [25].

Limited psychometric research has been conducted in sub-Saharan Africa in spite of the SDS being implemented in a notably different language, population, and region from which it was originally developed [24, 25]. A valid measure of social desirability could help us better statistically control for social desirability bias, particularly in similar resource-limited settings where self-reported measures continue to be the most cost- and resource-effective [26]. To address this knowledge gap, we conducted an exploratory study assessing certain psychometric properties of the 28-item Runyankole-translated Marlowe-Crowne SDS among PWH in Uganda. We also examined the association between social desirability and self-reported alcohol use in this setting.

## Methods

### Study design and population

This analysis used pooled baseline data from studies in Uganda, namely the Drinkers’ Intervention to Prevent Tuberculosis (TB, DIPT, Clinical Trials Registry NCT03492216 [Date of Registration: May 2018]) randomized controlled trial (RCT) [27], the Mobile Technology to Extend Clinic-Based Counseling for HIV + s in Uganda (Extend, Clinical Trials Registry NCT03928418 [Date of Registration: April 2019]) RCT [28], and the Alcohol Drinkers’ Exposure to Preventive Therapy for TB (ADEPT-T) observational study [29].

The DIPT RCT was conducted among heavy alcohol users with HIV and TB from 2018 to 2021 and aimed to evaluate the efficacy of incentive-based approaches to decrease alcohol use and improve isoniazid medication adherence (*N* = 680) [27, 30]. Recruitment and screening procedures are published in detail elsewhere [27, 30]. Enrolled participants received a 6-month course of isoniazid and pyridoxine (B6) and brief alcohol and medication adherence counseling based on Uganda’s standard of care. Participants underwent 1:1:1:1 randomization to one of the following interventions: no incentives (Arm 1, control), financial incentives for no recent alcohol use (Arm 2), financial incentives for recent isoniazid medication adherence (Arm 3), or financial incentives for decreasing alcohol use and increasing isoniazid medication adherence (Arm 4). Participants completed questionnaires and provided blood and urine samples, with data collection occurring at baseline and follow-up visits.

The Extend RCT evaluated the efficacy of counseling interventions to reduce alcohol use and increase HIV viral suppression among PWH who self-reported unhealthy alcohol use (*N* = 272) from 2019 to 2020, with screening procedures published elsewhere [28]. Eligible participants underwent 1:1:1 randomization to one of three study arms: brief advice based on Uganda’s standard of care (Arm 1, control), in-person counseling with interim boosters delivered by phone by the counsellor (Arm 2), or in-person counseling with interim boosters delivered by two-way automated systems (short messaging services [SMS] or interactive voice response [IVR], Arm 3). Participants completed questionnaires and provided blood samples at baseline and follow-up visits.

The ADEPT-T study was conducted between 2017 and 2020 and aimed to determine the incidence of isoniazid-related toxicity among PWH who consume alcohol and whether the rate of isoniazid-related toxicity varied by quantity of alcohol consumption (*N* = 301) [29]. Details pertaining to recruitment and screening procedures have been published elsewhere [29]. Participants were provided a 6-month course of isoniazid and pyridoxine (B6), completed questionnaires and provided blood samples at baseline and follow-up visits.

Participants provided written informed consent, and all study procedures were approved by the Ugandan National Council for Science and Technology, the Mbarara University of Science and Technology Research Ethics Committee, the Makerere University School of Medicine Research and Ethics Committee, and the institutional review boards (IRB) at the University of California San Francisco, Boston University, and Boston Medical Center.

### Data collection

In each of the studies, a third-party native Runyankole-speaker forward translated the survey from English to Runyankole. Ugandan study staff who were fluent in both Runyankole and English then back translated the survey from Runyankole to English, and discussed translations that were unclear with native English-speaking study staff based in the United States. Native Runyankole-speaking interviewers administered the baseline survey to study participants in either Runyankole or English, depending on the participant’s preference. The survey collected data on social desirability, alcohol use, religiosity, and other variables (e.g., sociodemographics). These data were measured consistently across studies using the same survey questions.

#### Social desirability

We used the shortened 28-item Marlowe-Crowne SDS (which had previously been used in sub-Saharan Africa) [24] rather than the 33-item scale because our Ugandan research colleagues agreed that the items omitted from the original 33-item SDS [21] would not be as relevant to individuals in Uganda and also to minimize respondent burden. We measured social desirability using binary response options (i.e., true, false, Table 1), and calculated a summed score with higher scores indicating greater levels of social desirability (range = 0–28); the 14-item Denial and 14-item Attribution Domains each had a range of possible scores from 0–14.

#### Alcohol Use

We assessed alcohol consumption using both self-reported and biomarker-measured data. We assessed self-reported alcohol use in the past 3 months using the 3-item Alcohol Use Disorders Identification Test—Consumption (AUDIT-C) [31]. We calculated a summed score, with higher scores indicating more alcohol use (range = 0–12). We also measured alcohol use with an alcohol biomarker, phosphatidylethanol (PEth), a blood-based biomarker of past month alcohol use [32–34]. Higher PEth values (ng/mL) correspond to more alcohol consumption in the past month.

**Table 1:** English and Runyankole-translated items from the shortened 28-item Marlowe-Crowne Social Desirability Scale

Item (Domain)	English (original)	Runyankole (translated)
1 (D)	It is sometimes hard for me to go on with my work if I am not encouraged	Obumwe nobumwe nikingumira kugumizamu nemirimo yangye naba ntatungire obushagiki
2 (D)	I sometimes feel resentful when I don’t get my way	Obumwe nimpurira nayeyanga naba ntakozire ekindikwenda
3 (D)	On a few occasions, I have given up doing something because I thought too little of my ability	Ahamirundi mikye etari mingi, mpanikire kugira ekinakora kyona ahabwokugira ngu nkateekateeka ngu tindikukibaasa
4 (D)	There have been times when I felt like rebelling against people in authority even though I knew they were right	Hariho emirundi emwe obunahuriire ninyenda ngu nyediimire abantu abari omubushoborozi nobu ndaabe nabeire nimanya ngu bahikire
5 (A)	No matter who I’m talking to, I’m always a good listener	Tikiine kakwate yaaba ngamba nooha nari oha, ndi omuhurikiza murungi
6 (D)	There have been occasions when I took advantage of someone	Habeireho obwire obu nyejuniise ondiijo muntu kwetungiramu omugasho nkomuntu
7 (A)	I’m always willing to admit it when I make a mistake	Buriijo naba ndi omunshobi ningikiriza
8 (D)	I sometimes try to get even rather than forgive and forget	Obumwe nobumwe ningyezaho kuhoora enzigu, okukira okusaasira nanokwebwa
9 (A)	I am always courteous, even to people who are disagreeable	Buriijo nimpa abantu eifubo, nangwa nobubakuba abo abantu abatarikwikirizana
10 (A)	I have never been irked when people expressed ideas very different from my own	Tinkagiraga kiniga abantu bahayo ebiteekateeko byabo biine entaniso mpango nebyangye
11 (D)	There have been times when I was quite jealous of the good fortune of others	Hariho emirundi obunagirira eihari emigisha mirungi yabandi
12 (D)	I am sometimes irritated by people who ask favors of me	Obumwe nishunguharira abantu abarikwenda ngu mbahwere
13 (A)	I have never deliberately said something that hurt someone’s feelings	Tinkagambaga kintu kyona ngyendereire kutoneka ebiteekateeko by’omuntu
14 (A)	I never hesitate to go out of my way to help someone in trouble	Tikirikuntwarira bwire kuruga omubyangye ebinaba ninkora okugira ngu mpwere ondiijo muntu ori omuburemeezi
15 (A)	I have never intensely disliked anyone	Tinkangaga muntu weena ekirenga
16 (D)	On occasion I have had doubts about my ability to succeed in life	Bumwe, nkagira okubanganisa omukubaasa kwangye kusingura omumagara gensi egi
17 (A)	I am always careful about my manner of dress	Ninkira kwegyendesereza munonga omumijwarire yangye
18 (D)	I like to gossip at times	Obumwe ninkira kugambukana / kukunda orugambo
19 (D)	I can remember “playing sick” to get out of something	Nimbaasa kwijuka “kwerwaza” okugira ngu ndugye omuburemeezi
20 (A)	I always try to practice what I preach	Ninkira kugyezaho kuta omunkora ebindikugamba
21 (A)	I don’t find it particularly difficult to get along with loud mouthed, obnoxious people	Tindikukira kukitungamu buzibu okukwatagana nabantu abarikugamba busha, nabo abatari kwemerwa
22 (A)	When I don’t know something I don’t at all mind admitting it	Kundikuba ntarikumanya kintu, tindikufayo nakakye kwikiriza ngu tindikukimanya
23 (D)	At times I have really insisted on having things my own way	Hariho obwire obunyangisirize ngu ebintu bikorwe nkokundikwenda
24 (D)	There have been occasions when I felt like smashing things	Hariho emirundi emwe obumpuriire ninyenda ngu nyate ebintu
25 (A)	I would never think of letting someone else be punished for my wrongdoings	Tinkagiraga ekyetengo kyokugira ngu omuntu ondiijo afubirwe ahabwenshobi zangye
26 (A)	I never resent being asked to return a favor	Tindikufayo kugira ngu nanye mpwere nkokunahwereirwe
27 (A)	I have never felt that I was punished without cause	Tinkahuriraga okugira ngu nafubirirwa busha nteine mushango / orubanja
28 (D)	I sometimes think when people have a misfortune they only got what they deserved	Heine obundikuteekateeka ngu abantu kubarikugira ebigwererezi nibaba bahoorwa

Response options in English: true, false

Translated response options in Runyankole: namazima, tikihikire

*Abbreviations*: *A* Attribution Domain of the SDS where responding “true” would be the socially desirable response, *D* Denial Domain of the SDS where responding “false” would be the socially desirably response

#### Religiosity

To measure religiosity, we used the 5-item Duke University Religion (DUREL) Index which measures religious involvement across three domains (i.e., organizational religious activity, non-organizational religious activity, and intrinsic religiosity) [35, 36]. The Organizational Religious Activity and Non-Organizational Religious Activity Domains consisted of a single item with 6-point Likert-type responses. The Intrinsic Religiosity Domain contained the remaining three items and used 5-point Likert-type responses. We calculated a summed score for this last domain, with higher scores indicating greater levels of intrinsic religiosity (range = 0–15). We assessed each DUREL Index domain separately as suggested by the developers of the scale [35].

#### Other covariates

We collected sociodemographic data on age (years), gender (men or women), and highest year of education. We measured social support using a modified 11-item version of the Duke University-University of North Carolina Social Support Scale [37]. We measured depression using the summed scores from the 20-item Center for Epidemiological Studies Depression (CESD) Scale [38]. Participants also provided biological specimens, including assessments of HIV viral load.

### Statistical analysis

Of the participants with baseline data across each of the three studies (*N* = 1250), our pooled analysis was restricted to those with complete social desirability data in Runyankole (*N* = 1153). We excluded individuals who completed the survey in English (*n* = 55) because phrases can have different meanings or interpretations in other languages [39] and our key objective was to assess the performance of the SDS specifically in Runyankole.

#### Sample characteristics

We calculated descriptive statistics (i.e., means [standard deviation, SD] or medians [interquartile range, IQR; and minimum to maximum range] for continuous variables and proportions for categorical variables) to characterize the study population in the pooled sample overall and stratified by study.

#### Construct validity

As previously mentioned, the Marlowe-Crowne SDS was designed to measure social desirability by assessing two components related to social desirability, namely, the tendency to avoid disapproval (captured by items in the Denial Domain) and the tendency to seek approval (captured by items in the Attribution Domain) [3, 21]. We used confirmatory factor analysis to assess whether the SDS had an underlying two-factor structure as designed, specifically by confirming whether items designed to measure avoiding disapproval loaded strongly onto the Denial Domain and whether items designed to measure seeking approval loaded strongly onto the Attribution Domain as designed [39]. We used the *cfa* function in the *lavaan* package in R [40] to generate diagonally weighted least square standard parameter estimates. We used several model fit indices to determine whether a model was a good fit for the data: Root Mean Square Error of Approximation (RMSEA, 0.01 = excellent fit, 0.05 = good fit, 0.08 = mediocre fit), Comparative Fit Index (CFI, > 0.90 indicative of good fit) and Tucker-Lewis Index (> 0.90 indicative of good fit) [41].

#### Internal consistency

We assessed the internal consistency of the SDS using Cronbach’s alpha [42] (via the *omega* function in the *psych* package in R [43]), given its widespread use in the literature and to allow for comparison with other studies. Cronbach alpha values above 0.70 are considered indicative of good internal consistency [39, 44], although we were cautious to interpret our findings strictly from these cutoffs given the known limitations of Cronbach’s alpha and its sensitivity to assumption violations that lead to upwardly or downwardly biased reliability estimates [44, 45]. To better understand the Cronbach’s alpha correlation coefficient, we plotted the reliability function to assess the alpha value along the spectrum of SDS scores (instead of a single point estimate averaging the correlation coefficient across all SDS scores; Supplementary Fig. 2). We also assessed internal consistency using McDonald’s omega total [46, 47] (via the *omega* function in the *psych* package in R [43]) which is considered a more robust measure of reliability [44, 48].

#### Item Performance

We used option characteristic curves (OCCs) to determine how well each item was performing by examining each item’s discrimination and difficulty [39, 49]. An item’s discrimination assesses how well that particular item differentiates people with different levels of social desirability. Items with high discrimination (i.e., response curves with steep slopes) are more precise than those with low discrimination. An item’s difficulty estimates the location along the continuum of social desirability where there is more than a 50% probability of endorsing that item. To endorse a difficult item (i.e., one with a location parameter towards the right or higher end of the social desirability continuum), an individual would need to have high levels of social desirability whereas they would need little social desirability to endorse an easy item. In a sample of participants with ranging social desirability levels, many participants would likely endorse easier items but fewer would endorse difficult items. As such, scales with items ranging in difficulty are better able to assess and parse out which individuals have higher levels of social desirability. We estimated OCCs using the *ksIRT* function in the *KernSmoorthIRT* package in R [50].

#### Differential item functioning by gender

We assessed differential item functioning (DIF) by gender, which is a way to detect differential measurement error. Assessing DIF by gender allows us to detect whether participants with the same level of social desirability responded differently to specific items due to their gender. DIF analysis assumes unidimensionality, so we assessed DIF within each SDS domain. Likelihood ratio tests were employed, where a final anchored model estimated the magnitude of significant DIF across items. This allowed us to determine whether there were any significant differences in item responses in each domain, as well as whether any significant differences were also of large magnitude [51]. We then plotted expected scale scores to assess the impact of any notable DIF on the scale overall [52].

#### Concurrent validity

We assessed the correlations between the SDS and each domain of the DUREL religiosity index (as recommended by its developers [35, 36]) using the *pwcorr* statement in STATA. We evaluated whether correlation coefficients were weak (r < 0.3), moderate (*r* = 0.4 – 0.6), or strong (*r* > 0.7) [53].

#### Exploratory assessment of the association between SDS and self-reported alcohol use

We assessed the association between the Runyankole-translated SDS and self-reported alcohol use to determine whether the results were consistent with previous work in Uganda [25]. We first conducted regression diagnostics and found that there were no major violations to the linear regression assumptions of linearity, homoscedasticity, and normality. We then conducted linear regression modeling AUDIT-C summed scores as a function of SDS summed scores with a random effect to account for variability between studies. In our adjusted model, we additionally adjusted for age, gender, education, depression, religiosity, and biomarker-measured alcohol use (i.e., PEth). These covariates were identified a priori from expert knowledge, and associations between these variables (i.e., age [54–57], gender [57–60], education [57, 61], depression [62–64], and religiosity [65–69]), social desirability, and alcohol use have been documented in the literature. We also adjusted for the alcohol biomarker PEth to mitigate potential measurement issues in self-reported alcohol use assessments which are prone to underreporting [70–72]. Although we theorized that social support could confound the relationship between social desirability and self-reported alcohol use given its association with both [73–75], we did not include this covariate in our adjusted models as this variable was not collected in all of the studies. We also explored the association between SDS summed scores and self-reported alcohol use stratified by study (provided in Supplementary Material).

We conducted our analyses using STATA/SE 17.0 for Windows or R (Version 4.3.1) [76]. We assumed a two-sided *p* < 0.05 to be statistically significant.

## Results

### Sample characteristics

In our pooled baseline sample of PWH (*N* = 1153), participants had a mean age of 40.42 years (standard deviation [SD] = 9.89), 63% were men, 64% reported some primary school education, and most of the sample (91%) had an undetectable HIV viral load (< 40 copies/mL, Table 2). Participants self-reported a mean AUDIT-C score of 5.55 (SD = 3.12), while the median PEth concentration was 190 ng/mL (IQR = 34–474 ng/mL). As expected, given their differing study eligibility criteria, participants in ADEPT-T had lower PEth values compared to those in DIPT and Extend (medians of 15 ng/mL versus 264 ng/mL and 259 ng/mL, respectively). This corresponded with the trends in self-reported alcohol use, with those in ADEPT-T self-reporting lower alcohol use (mean AUDIT-C = 2.77) compared to the participants in the other studies (mean AUDIT-C scores of 6.34 and 6.89 in DIPT and Extend, respectively).

**Table 2:** Sample characteristics of persons with HIV in Uganda overall and by study, n(%)

Characteristics	Overall (*N* = 1153)	Study		
		DIPT (*n* = 599)	Extend (*n* = 261)	ADEPT-T (*n* = 293)
Age (years)				
Med (IQR)	40 (32–47)	39 (32–47)	40 (33–46)	40 (33–47)
Mean (SD)	40.42 (9.89)	40.40 (10.18)	40.24 (9.49)	40.61 (9.68)
Men	721 (63)	408 (68)	169 (65)	144 (49)
Highest level of education				
None	141 (12)	99 (17)	14 (5)	28 (10)
Some primary school	733 (64)	387 (65)	160 (61)	186 (63)
Some secondary school	211 (18)	92 (15)	61 (23)	58 (20)
Vocation school	61 (5)	20 (3)	24 (9)	17 (6)
University	7 (1)	1 (< 1)	2 (1)	4 (1)
Undetectable HIV viral load	1021 (91)	525 (90)	234 (90)	262 (92)
Recent alcohol use				
Self-reported AUDIT-C				
Med (IQR)	5 (3.5–8)	6 (4–8)	7 (4–9)	2 (0–4)
Mean (SD)	5.55 (3.12)	6.34 (2.45)	6.89 (2.82)	2.77 (2.88)
Biomarker-measured PEth (ng/mL)				
Med (IQR)	190 (34–474)	264 (93–583)	259 (68–520)	15 (1–188)
Mean (SD)	342.07 (426.01)	416.75 (457.41)	387.01 (438.16)	149.39 (257.07)
Depression (CESD)				
Med (IQR)	6 (1–13)	5 (1–10)	13 (6–28)	3 (0–10)
Mean (SD)	9.66 (11.60)	7.44 (8.69)	18.37 (15.48)	6.42 (8.63)
Religiosity (DUREL Index)				
Organizational religious activity				
Med (IQR)	4 (3–5)	4 (3–5)	4 (3–5)	5 (4–5)
Mean (SD)	3.90 (1.40)	3.78 (1.39)	3.85 (1.39)	4.18 (1.40)
Non-organizational religious activity				
Med (IQR)	2 (1–5)	2 (1–4)	2 (1–4)	4 (1–5)
Mean (SD)	2.74 (1.67)	2.49 (1.63)	2.63 (1.59)	3.32 (1.69)
Intrinsic religiosity				
Med (IQR)	15 (12–15)	15 (11–15)	12 (12–15)	15 (14–15)
Mean (SD)	12.78 (3.29)	12.34 (3.91)	12.72 (2.19)	13.76 (2.41)

*Abbreviations*: *ADEPT-T* The Alcohol Drinkers’ Exposure to Preventive Therapy for TB Study, *AUDIT-C* Alcohol Use Disorders Identification Test – Consumption, *CESD* Center for Epidemiological Studies Depression, *DIPT* The Drinkers’ Intervention to Prevent Tuberculosis RCT, *DUREL* Duke University Religion Index, *Extend* The Mobile Technology to Extend Clinic-Based Counseling for HIV + s in Uganda RCT, *IQR* Interquartile range, *med* median, *PEth* Phosphatidylethanol

### 28-item Runyankole-translated SDS

The 28-item Runyankole-translated SDS had a mean summed score of 19.59 (SD = 3.51, range = 8–28), and a median score of 20 (IQR = 17–22). The 14-item Denial Domain had a mean summed score of 8.39 (SD = 3.32, range = 0–14) and a median score of 9 (IQR = 6–11); the 14-item Attribution Domain had a mean summed score of 11.20 (SD = 2.16, range = 0–14) and a median score of 11 (IQR = 10–13).

#### Construct validity and internal consistency

In our CFA of a two-factor structure, we determined that items had acceptable factor loadings in each domain (Denial Domain: 0.50 to 0.74, Attribution Domain: 0.34 to 0.76, Table 3) and multiple fit indices indicated a satisfactory model fit (RMSEA = 0.07, CFI = 0.84, TLI = 0.82). We found good internal consistency in the Denial Domain (Omega total [Ω_total_] of 0.82 and Cronbach’s α of 0.80) and satisfactory internal consistency in the Attribution Domain (Ω_total_ of 0.69 and Cronbach’s alpha of 0.63). Plotting the reliability function for the Attribution Domain revealed that Cronbach alpha values were high (~ 0.80) among individuals with Attribution Domain scores below the mean (i.e., 11.20), but lower among individuals with domain scores above the mean (Supplementary Fig. 2).

**Table 3:** Construct validity and internal consistency of the Runyankole-translated Marlowe-Crowne Social Desirability Scale among persons with HIV in Uganda

	28-item SDS	27-item SDS
Construct validity via confirmatory factor analysis		
Covariance between factors	-0.34	-0.28
Factor loadings range		
Denial Domain	0.50 to 0.74	0.50 to 0.72
Attribution Domain	0.34 to 0.76	0.42 to 0.80
Model fit indices		
RMSEA	0.07	0.06
CFI	0.84	0.86
TLI	0.82	0.85
Internal consistency		
Omega’s total		
Denial Domain	0.82	0.82
Attribution Domain	0.69	0.72
Cronbach’s alpha		
Denial Domain	0.80	0.80
Attribution Domain	0.63	0.67

*Abbreviations*: *CFI* Comparative Fit Index, *RMSEA* Root Mean Square Error of Approximation, *TLI* Tucker-Lewis Index, *SDS* Social Desirability Scale

#### Item-level performance

**Fig. 1:**
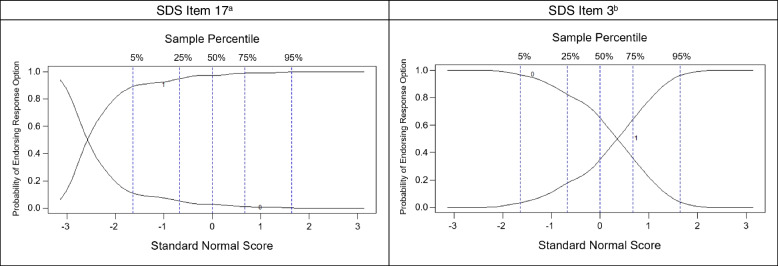
Option characteristic curves for representative items from the Runyankole-translated Marlowe-Crowne SDS. Abbreviations: SDS = Social Desirability Scale. ^a^ Item 17 (“I am always careful about my manner of dress”) is part of the Attribution Domain of the SDS. Responding “true” to Item 17 indicates more social desirability. ^b^ Item 3 (“On a few occasions, I have given up doing something because I thought too little of my ability”) is part of the Denial Domain of the SDS. Responding “false” to Item 3 indicates more social desirability

**Fig. 2:**
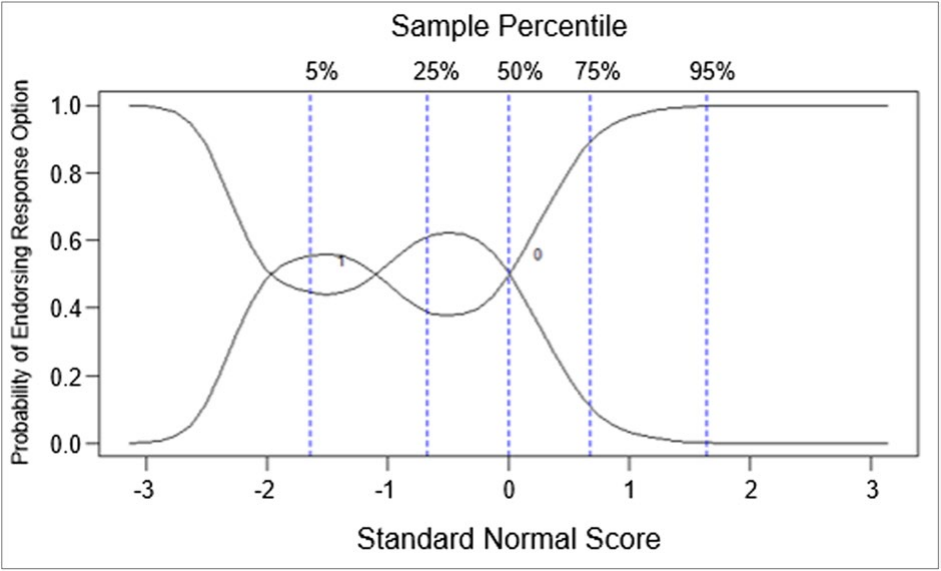
Option characteristic curve depicting performance issues in Item 14^a^ from the Runyankole-translated Marlowe-Crowne Social Desirability Scale. ^a^ Item 14 (“I never hesitate to go out of my way to help someone in trouble”) is part of the Attribution Domain of the SDS. Responding “true” to Item 14 indicates more social desirability

Most items had high discrimination, as depicted by the steep slopes of the response curves in Fig. 1. We also observed that most items had low or moderate difficulty in our sample. We observed that Item 14 (“I never hesitate to go out of my way to help someone in trouble”) had certain performance issues given the undulating slopes along the x-axis of the response curves (Fig. 2). OCCs for all items are presented in Supplementary Fig. 1.

#### Differential item functioning by gender

We did not find any notable DIF by gender in the Denial Domain, as indicated by the approximately overlapping slopes of expected test scores between men and women (Supplementary Table 3). However, we detected DIF by gender in the Attribution Domain (Table 4), and found that one particular item (i.e., Item 14) was driving this measurement error considering the significance and associated magnitude of its detected DIF (*p*-value = 0.002, DIF effect size = 0.678, Table 4). Given that this was the same problematic item that was identified when assessing item-level performance with OCCs, we removed it from the scale to mitigate the measurement error.

**Table 4:** Assessing differential item functioning (DIF) by gender in the Attribution Domain of the Runyankole-translated Marlowe-Crowne Social Desirability Scale

14-item Attribution Domain			
Item	*P*-value	DIF Effect Size	Expected Test Scores
5	0.144	-0.025	
7	0.444	0.004	
9	0.291	-0.163	
10	0.011	-0.329	
13^a^	-	-	
14	0.002	0.678	
15	0.783	-0.189	
17	0.836	-0.006	
20	0.960	0.242	
21	0.421	-0.193	
22	0.056	-0.053	
25^a^	-	-	
26	< 0.001	0.221	
27	0.502	-0.061	
Modified 13-item Attribution Domain^b^			
Item	*P*-value	DIF Effect Size	Expected Test Scores
5	0.136	-0.020	
7	0.603	0.029	
9	0.343	-0.132	
10	0.023	-0.283	
13^a^	-	-	
15	0.535	-0.142	
17^a^	-	-	
20	0.945	0.250	
21	0.790	-0.216	
22	0.279	-0.078	
25	0.759	0.015	
26	0.006	0.297	
27	0.236	-0.017	

^a^Used as anchor item

^b^the Attribution Domain was modified from a 14-item subscale to a 13-item subscale by removing problematic Item 14

### Modified 27-item Runyankole-translated SDS

The 27-item Runyankole-translated SDS had a mean summed score of 18.95 (SD = 3.50, range = 7–27), and a median score of 19 (IQR = 16–22). The 14-item Denial Domain remained unchanged and, as such, had the same mean and median scores as reported above. After removing one item from the 14-item Attribution Domain, the subsequent 13-item Attribution Domain had a mean summed score of 10.56 (SD = 2.00, range = 0–13) and a median score of 11 (IQR = 10–12).

#### Construct validity and internal consistency

Excluding Item 14 seemed to slightly improve model fit in the CFA of a two-factor structure (RMSEA= 0.06, CFI = 0.86, TLI = 0.85, Table 3). Removing Item 14 (which was part of the Attribution Domain) slightly improved the internal consistency within the Attribution Domain (Ω_total_ = 0.72 and Cronbach’s α = 0.67); as expected, the internal consistency coefficients from the Denial Domain remained unchanged given that no modifications were made to this domain (Ω_total_ = 0.82 and Cronbach’s alpha = 0.80).

#### Differential item functioning by gender

We did not detect significant, notable DIF by gender in the Denial or Attribution Domains of the modified 27-item SDS (Table 4).

#### Concurrent validity

The modified 27-item SDS was weakly correlated (Pearson correlation coefficient = 0.08, *p* < 0.01) with the Organizational Religious Activity Domain of the DUREL religiosity index, as was the SDS’s 14-item Denial Domain (Pearson correlation coefficient = 0.07, *p* = 0.01, Table 5). The 27-item SDS’s Attribution Domain was weakly correlated (*r* = 0.07, *p* = 0.02) with the DUREL’s Intrinsic Religiosity Domain.

**Table 5:** Concurrent validity between the modified 27-item Runyankole-translated Marlowe-Crowne Social Desirability Scale and the DUREL Index among persons with HIV in Uganda

	Modified 27-item SDS		14-item Denial Domain		Modified 13-item Attribution Domain	
	Pearson correlation coefficient (r)	*p*-value	Pearson correlation coefficient (r)	*p*-value	Pearson correlation coefficient (r)	*p*-value
DUREL Index						
Organizational Religious Activity	0.08	< 0.01	0.07	0.01	0.03	0.34
Non-Organizational Religious Activity	0.00	0.90	-0.02	0.57	0.03	0.24
Intrinsic Religiosity	0.02	0.48	-0.02	0.51	0.07	0.02

*Abbreviations*: *DUREL* Duke University Religion Index, *SDS *Social Desirability Scale

#### Exploratory assessment of the association between the modified SDS and self-reported alcohol use

We found significant inverse associations between the 27-item SDS and self-reported alcohol use in our unadjusted (β = -0.08, 95% confidence interval [CI]: -0.13 to -0.04, *p*-value < 0.01) and adjusted (β = -0.05, 95% CI: -0.09 to -0.01, *p*-value = 0.03) mixed effects models (Table 6). Findings pertaining to these associations stratified by study are presented in Supplementary Table 4.

**Table 6:** Mixed effects^a^ linear regression of the association between the modified 27-item Runyankole-translated Marlowe-Crowne Social Desirability Scale and self-reported alcohol use

	Unadjusted			Adjusted^b^		
	β	95% CI	*p*-value	β	95% CI	*p*-value
Self-reported alcohol use (AUDIT-C)	-0.08	-0.13 to -0.04	< 0.01	-0.05	-0.09 to -0.01	0.03

*Abbreviations*: *AUDIT-C* Alcohol Use Disorders Identification Test – Consumption, *β* beta coefficient, *CI* confidence interval

^a^Both the unadjusted and adjusted models include a random effect to account for variability between studies

^b^Adjusted for age, gender, education, depression, religiosity, and the alcohol biomarker phosphatidylethanol

## Discussion

We found that the 28-item Runyankole-translated SDS had satisfactory construct validity and internal consistency in our sample of PWH in Uganda, but discovered through further psychometric assessment that one problematic item (Item 14) was driving significant measurement error between men and women. Although removing this problematic item only marginally improved the scale’s construct validity and internal consistency, it attenuated the differential measurement bias that we detected which lends justification for using the modified 27-item SDS in this setting. Our study adds to the limited research regarding the validity and reliability of the SDS in Uganda given that we are the first to report the translated scale’s construct validity, the performance of individual items, differential item functioning by gender, and concurrent validity.

Our item-level analysis revealed a problematic item (Item 14) with subpar discrimination and difficulty, which was later identified as a key driver of differential measurement error by gender in one of the scale’s domains. Native Runyankole-speaking members of our team in Uganda provided important clarification that there may have been some translation issues with this particular item, which may have affected its performance in our sample. Through their feedback, we identified that the item in Runyankole was more translatable to “it does not take me a lot of time to stop what I am doing and help someone,” which likely altered some of the intended meaning from the original item in English (“I never hesitate to go out of my way to help someone in trouble”). The issues that we identified at the item- and scale-level justified our decision to remove Item 14 from the scale and proceed with a modified 27-item version of the translated scale. The mistranslation of Item 14 may have led to differential responses between women and men due to the subtle differences in meaning and the cultural and gender norms that are prevalent in Uganda [77–81]. The original item assesses whether someone would act “to help someone in trouble,” which is relatable to a more pressing situation where an individual might intervene to come to the aid of someone in a dire situation; this scenario may evoke a more consistent response between women and men who understand that the socially desirable response would be to always help someone in an urgent or heightened difficult situation. The subtle difference in phrasing of the Runyankole translation of Item 14, on the other hand, speaks more to an individual’s altruism in pausing their own routine (“it does not take me a lot of time to stop what I am doing…”) and support someone else (“… to help someone”); this may have been interpreted as supporting someone with something less urgent, given that people can need help for many things including minor, nonurgent tasks. Given the sociocultural norms in Uganda suggesting that women should be benevolent, submissive, nurturing, caring, and willing to prioritize the needs of their family or their community above their own, it is possible that women in our sample responded in the affirmative to this question compared to men who do not face the same type of societal expectations [77–81].

The observed mean score (18.95) for the modified 27-item SDS in our sample was comparable after rescaling (re-scaled mean score = 9.11) to the median score of 9.0 in a 2011–2014 study in Uganda using the 13-item SDS Short Form C [25]. Confirmatory factor analysis of the modified 27-item Runyankole-translated SDS suggested satisfactory construct validity, including marginally improved model fit indices compared to the 28-item SDS. Removing Item 14 also somewhat improved the scale’s reliability, indicated by the increased internal consistency coefficients in the Attribution Domain of the modified 27-item SDS. The Omega’s total (Ω_Total_) coefficient for the modified 27-item SDS indicated satisfactory internal consistency, which is considered a more robust measure of reliability compared to Cronbach’s alpha [44, 48]. However, as Cronbach’s alpha is frequently cited in the literature, we further explored alpha internal consistency values in the Attribution Domain (which had a lower, average alpha value). We found fluctuations in internal consistency with better internal consistency among participants with less social desirability compared to the rest of our sample (i.e., those with scores below the mean) but lower internal consistency among participants with high levels of social desirability (i.e., those with scores above the mean). Most of our sample had high Attribution scores and Cronbach’s alpha is calculated partly as a function of the variance of individual items [42]. As such, it appears that there was low individual-item variance with which to differentiate the large proportion of our participants with high Attribution scores, which would have attenuated the alpha coefficient among this group of participants with high scores. This does not indicate that the Attribution Domain had poor internal consistency (particularly since the Omega total coefficient indicated good internal consistency), but may be more related to the method in which Cronbach’s alpha is calculated which can result in downwardly biased estimates. Future work assessing the internal consistency of the scale could consider implementing Likert-type response options (instead of the binary responses used in this study) or adding additional items to the scale, as both of these components could help to better differentiate individuals with high social desirability scores in this setting.

Using the modified 27-item SDS, we also found that social desirability was inversely associated with self-reported alcohol use (after adjusting for identified confounders including biomarker-measured alcohol use). Our findings are consistent with previous work in the same setting [25], and build on the other published evidence that social desirability is associated with misreporting [4–7].

This study had some limitations. Our study population was recruited via convenience sampling in healthcare clinics, which limits the generalizability of our findings. However, these methods were valuable to recruit a sufficient sample of our target population. Moreover, our study was not restricted to any particular setting; participants in our pooled sample were recruited across urban, semi-urban, and rural areas. We could not assess concurrent validity of the Marlowe-Crowne SDS with other social desirability measures because the studies in our pooled analysis were not designed for psychometric evaluation and these data were not collected. We assessed the concurrent validity of the SDS with religiosity, but the religiosity measure (i.e., DUREL) was not validated in Runyankole because its psychometric assessment was beyond the scope of our study. Also, we theorized that social support may confound the relationship between social desirability and self-reported alcohol use but did not adjust for this variable in our exploratory analyses because it was not collected in all of the studies. Despite these limitations, our study is a contribution to the limited psychometric research available pertaining to the Runyankole-translated SDS in this setting.

## Conclusions

Although the 28-item Runyankole-translated SDS demonstrated satisfactory construct validity and internal consistency, we recommend that researchers using the current Runyankole translation of the scale remove Item 14 given that doing so mitigated observed differential measurement error by gender. The modified 27-item Runyankole-translated SDS had satisfactory construct validity and internal consistency in our sample, and can facilitate our understanding of the role of social desirability in underreporting of stigmatized behaviors like alcohol consumption in this setting. In addition to the more generalized social desirability measured in the Marlowe-Crowne SDS, it may be more nuanced to assess social desirability in relation to specific behaviors (e.g., sexual partnerships, condom use) which is an area for further research. Future studies should continue to evaluate the psychometric properties of the Runyankole-translated SDS, including evaluating whether implementing Likert-type response options or adding items to the scale may improve its performance. Although we conducted forward and back translations of the Marlowe-Crowne SDS to produce an appropriate Runyankole translation of the scale, phrases can often have different meanings or interpretations in translated languages and it would be useful for future qualitative work (including focus groups with individuals proficient in English and Runyankole) to assess whether the translated scale fully captures the intended meaning in English across all scale items or if improvements can be made. Qualitative work would also be beneficial in specifically retranslating Item 14, followed by quantitative psychometric analyses reevaluating whether its performance has improved. Although outside of the scope of this analysis, future work should consider whether the SDS performs differently in various regions in Uganda given cultural and tribal differences across the country.

### Supplementary Information

Supplementary file 1.

## Data Availability

The dataset generated for these analyses are available on reasonable request from the senior author.

## Abbreviations

ADEPT-T: Alcohol Drinkers’ Exposure to Preventive Therapy for TB Study
AUDIT-C: Alcohol Use Disorders Identification Test—Consumption
CFI: Comparative Fit Index
DIF: Differential Item Functioning
DIPT: Drinkers’ Intervention to Prevent Tuberculosis RCT
DUREL: Duke University Religion Index
Extend: Mobile Technology to Extend Clinic-Based Counseling for HIV + s in Uganda RCT
IRB: Institutional Review Board
IVR: Interactive voice response
OCC: Option characteristic curve
PEth: Phosphatidylethanol
PWH: Persons with HIV
RMSEA: Root Mean Square Error of Approximation
SDS: Social Desirability Scale
TLI: Tucker-Lewis Index
