# Supplementary material for: Psychometric assessment of the Runyankole-translated Marlowe-Crowne Social Desirability Scale among persons with HIV in Uganda
Source: BMC Public Health. 2024 Jun 19;24:1628. doi: 10.1186/s12889-024-18886-z (PMC11186284; doi:10.1186/s12889-024-18886-z)
Supplement: Supplementary file 1 [file 12889_2024_18886_MOESM1_ESM.pdf]

Supplementary Material

Supplementary Figure 1. Option characteristic curves for all 28 items in the Runyankole-translated Marlowe-Crowne Social Desirability Scale

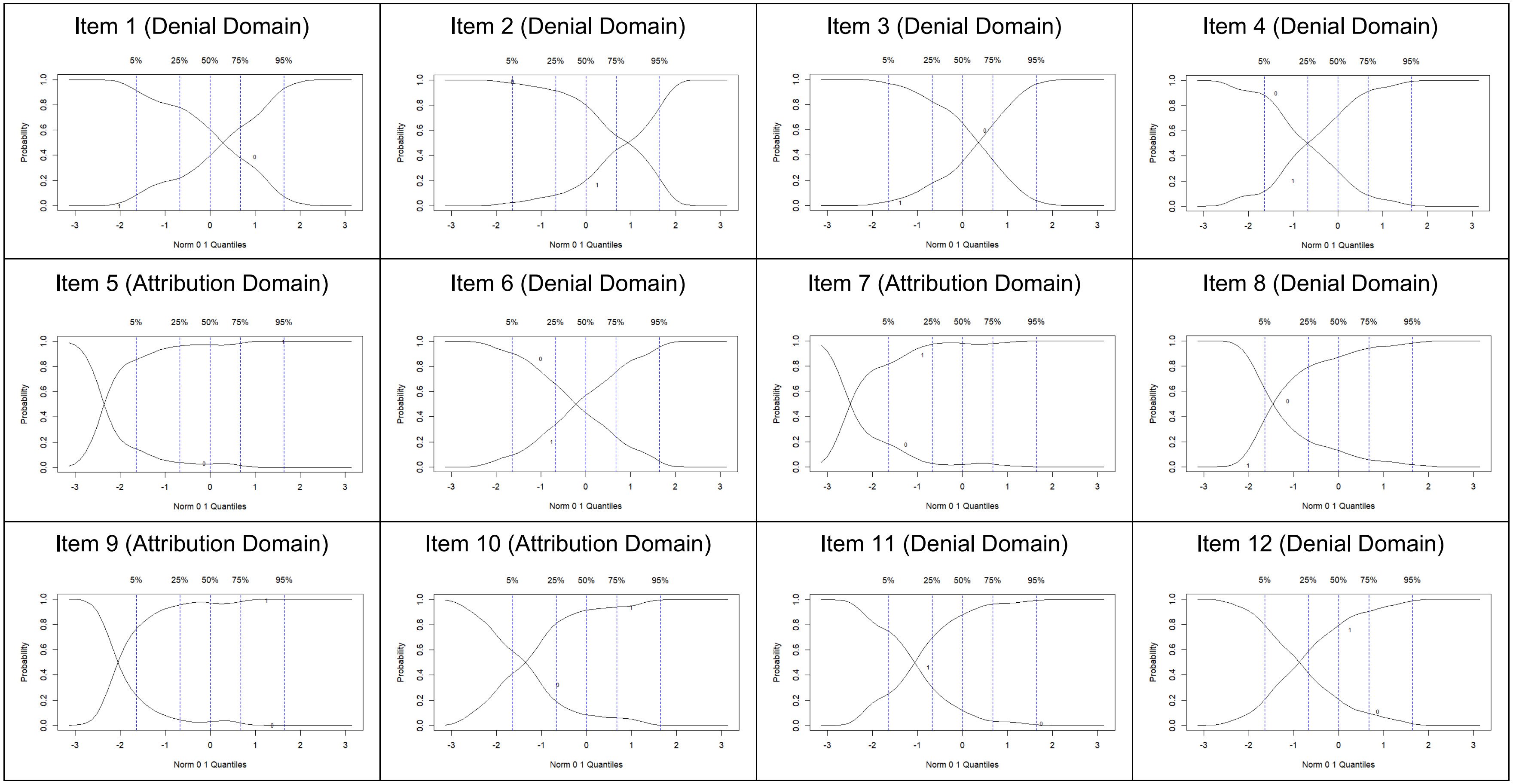

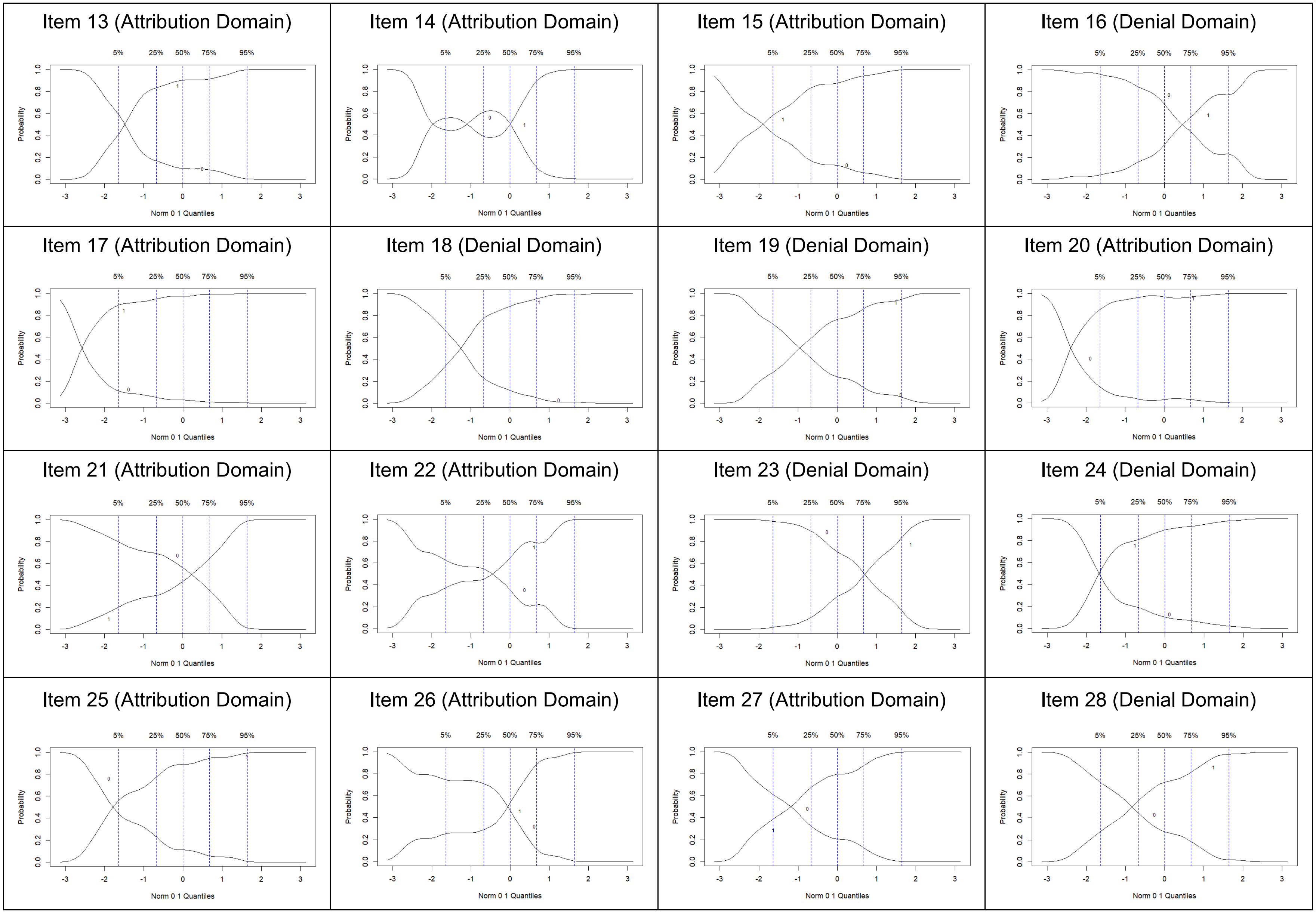
Item 13 (Attribution Domain)
Item 14 (Attribution Domain)
Item 15 (Attribution Domain)
Item 16 (Denial Domain)
Item 17 (Attribution Domain)
Item 18 (Denial Domain)
Item 19 (Denial Domain)
Item 20 (Attribution Domain)
Item 21 (Attribution Domain)
Item 22 (Attribution Domain)
Item 23 (Denial Domain)
Item 24 (Denial Domain)
Item 25 (Attribution Domain)
Item 26 (Attribution Domain)
Item 27 (Attribution Domain)
Item 28 (Denial Domain)
5% 25% 50% 75% 95%
Probability
Norm 0 1 Quantiles

Supplementary Figure 2. Reliability function for the 14-item Attribution Domain plotting Cronbach's alpha coefficient values along the spectrum of possible scores.

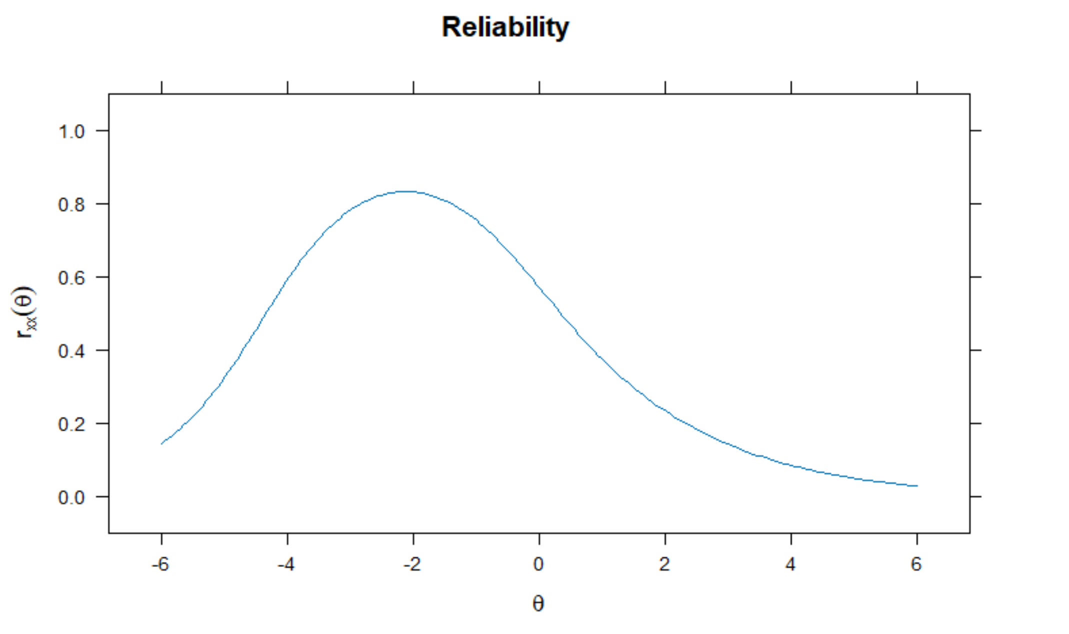

Supplementary Table 1. English and Runyankole-translated items from the adapted* Alcohol Use Disorders Identification Test – Consumption (AUDIT-C)

English (original)	Runyankole (translated)
In the past 3 months, how often did you have a drink containing alcohol?[a]	Nikangahi obu onyweire ekyokunywa kirimu ebirikusinza omumeezi 3 agahingwire?[b]
How many drinks containing alcohol did you have on a typical day when you were drinking in the past 3 months?[c]	Omumeezi 3 agahweire onyweire ebirikusinza birikwingana ki aha izooba rimwe obwobeire nonywa ebirikusinza?[d]
How often did you have six or more drinks on one occasion in the past 3 months?[e]	Nikangahi obu onyweire emisha mukaaga nokukiraho ahamurundi gumwe omumeezi 3 agahingwire?[f]

*The AUDIT-C was adapted to ask study participants to recall their drinking behavior in the past three months.
[a] Response options in English: never, monthly or less, 2 to 4 times per month, 2 to 3 times per week, 4 or more times per week
[b] Response options translated in Runyankole: tiburiho, buri kwezi nari ahansi yokwezi, emirundi 2 kuhitsya 4 buri kwezi, Emirundi 2 kuhitsya 3 buri sande, emirundi 4 nari okukiraho buri sande
[c] Response options in English: None, 1 or 2, 3 or 4, 5 or 6, 7 or 9, 10 or more
[d] Response options translated in Runyankole: tikiriho, 1 or 2, 3 or 4, 5 or 6, 7 or 9, 10 nokukiraho
Response options in English: never, less than monthly, monthly, weekly, daily or almost daily
[f] Response options translated in Runyankole: tiburiho, ahansi yokwezi, buri kwezi, buri sande, buri eizoba nari haihi buri eizoba

Supplementary Table 2. English and Runyankole-translated items from the adapted Duke University Religion (DUREL) Index

English (original)	Runyankole (translated)
Organizational Religious Activity Domain	
How often do you attend church, mosque, or other religious meetings?[a]	Nikangahi obu orikuza omukanisa / omukereziya, omumuzigiti, nari ezindi nteerane zediini?[b]
Non-Organizational Religious Activity Domain	
How often do you spend time in private religious activities, such as prayer, meditation or Bible/Koran study?[c]	Nikangahi obu orikumara obwire oyehereire omubikorwa byediini, nka okushaba,okushoma ebitabo ebirikwera nka Baibuli / Koran?[d]
Intrinsic Religiosity Domain	
In my life, I experience the presence of the Divine (i.e., God)[e]	Omumagara gangye, ninyehuriramu obwa Rukiraboona (Ruhanga)[f]
My religious beliefs are what really lie behind my whole approach to life[e]	Enyikiriza zangye omudiini nibyo birikutuma okundikutwara ensi[f]
I try hard to carry my religion over into all other dealings in life[e]	Ningyezaho nkokukirikubaasika kureeba ngu ediini yangye nagitwara omubintu byoona ebindikukora omumagara gangye[f]

[a] Response options in English: never, once a year or less, a few times a year, a few times a month, once a week, more than once per week
[b] Response options translated in Runyankole: tiburiho, omurundi gumwe omumwaka nari ahansi yomurundi gumwe, emirundi mikye omumwaka, emirundi mikye omukwezi, omurundi gumwe omusande, okurenga ahamurundi gumwe omusande
[c] Response options in English: rarely or never, a few times a month, once a week, two ormore times per week, daily, more than once a day
[d] Response options translated in Runyankole: nibukye munonga / tiburiho, emirundi mikye omukwezi, omurundi gumwe omusande, emirundi ebiri nari okukiraho omusande, buri izooba, okurenga ahamurundi gumwe omwizooba
[e] Response options in English: definintely not true, tends not to be true, unsure, tends to be true, definintely true of me
[f] Response options translated in Runyankole: buzimazima tikihikire, nikishushana nkekitahikire, tinakikwasagye, nikishusha nkekihikire, buzimazima kihikire ahabwangye

Supplementary Table 3. Assessing differential item functioning (DIF) by gender in the denial domain of the 28-item Runyankole-translated Marlowe-Crowne Social Desirability Scale.

Item	p-value	DIF Effect Size
1	0.548	0.151
2[a]	-	-
3	0.008	-0.170
4	0.660	-0.025
6	0.001	0.109
8	0.650	-0.239
11	0.180	-0.168
12	0.293	-0.019
16	0.753	-0.364
18	0.217	-0.193
19	0.025	0.147
23[a]	-	-
24	0.254	-0.293
28	0.600	-0.115

Expected Test Scores

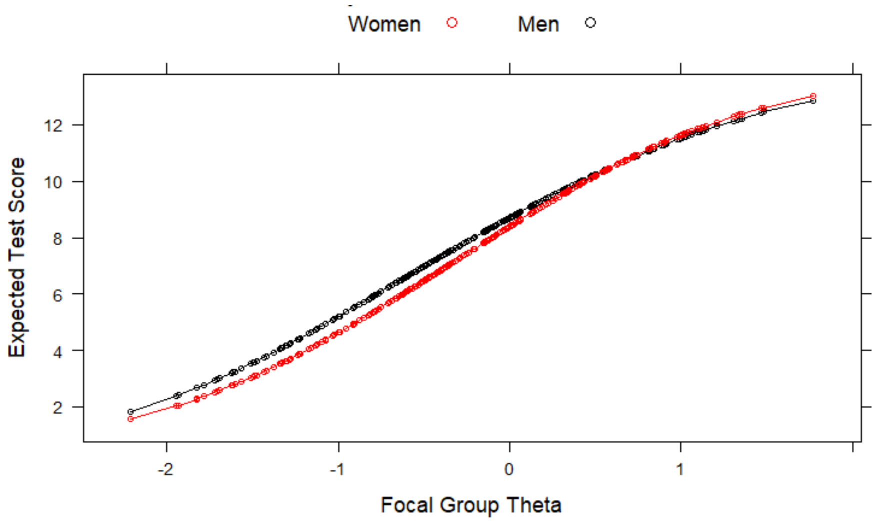

[a] Used as anchor item

Supplementary Table 4. Linear regression of the association between the modified 27-item Runyankole-translated Marlowe-Crowne Social Desirability Scale and self-reported alcohol use stratified by study

	Study		
	DIPT (n=599)	Extend (n=261)	ADEPT-T (n=293)
Unadjusted Model			
Self-reported alcohol use β	-0.10	-0.02	-0.10
95% CI	-0.15 to -0.04	-0.13 to 0.08	-0.21 to 0.01
p-value	<0.01	0.67	0.07
Adjusted[a] Model			
Self-reported alcohol use β	-0.08	-0.01	-0.07
95% CI	-0.13 to -0.03	-0.12 to 0.10	-0.16 to 0.02
p-value	<0.01	0.88	0.12

Abbreviations: ADEPT-T= the Alcohol Drinkers' Exposure to Preventive Therapy for TB study, AUDIT-C= Alcohol Use Disorders Identification Test – Consumption, β= beta coefficient, CI= confidence interval, DIPT= the Drinkers' Intervention to Prevent Tuberculosis RCT, Extend= the Mobile Technology to Extend Clinic-Based Counseling for HIV+s in Uganda RCT

[a] Adjusted for age, gender, education, depression, religiosity, and the alcohol biomarker phosphatidylethanol.
